# An Automated Clubbed Fingers Detection System Based on YOLOv8 and U-Net: A Tool for Early Prediction of Lung and Cardiovascular Diseases

**DOI:** 10.3390/diagnostics14192234

**Published:** 2024-10-07

**Authors:** Wen-Shin Hsu, Guan-Tsen Liu, Su-Juan Chen, Si-Yu Wei, Wei-Hsun Wang

**Affiliations:** 1 Department of Medical Information, Chung Shan Medical University, Taichung 402201, Taiwan; wshsu@csmu.edu.tw (W.-S.H.);; 2 Informatics Office Technology, Chung Shan Medical University Hospital, Taichung 402201, Taiwan; 3 Department of Public Health, Chung Shan Medical University, Taichung 402201, Taiwan; 4 College of Medicine, National Chung Hsing University, Taichung 402202, Taiwan; 5 Department of Golden-Ager Industry Management, Chaoyang University of Technology, Taichung 413310, Taiwan; 6 Department of Orthopedic Surgery, Changhua Christian Hospital, Changhua 500209, Taiwan; 7 Department of Medical Imaging and Radiology, Shu-Zen Junior College of Medicine and Management, Kaohsiung 82144, Taiwan

**Keywords:** lung diseases, cardiovascular diseases, clubbed fingers detection, YOLOv8, U-Net, cloud computing, real-time detection

## Abstract

**Background/Objectives**: Lung and cardiovascular diseases are leading causes of mortality worldwide, yet early detection remains challenging due to the subtle symptoms. Digital clubbing, characterized by the bulbous enlargement of the fingertips, serves as an early indicator of these diseases. This study aims to develop an automated system for detecting digital clubbing using deep-learning models for real-time monitoring and early intervention. **Methods:** The proposed system utilizes the YOLOv8 model for object detection and U-Net for image segmentation, integrated with the ESP32-CAM development board to capture and analyze finger images. The severity of digital clubbing is determined using a custom algorithm based on the Lovibond angle theory, categorizing the condition into normal, mild, moderate, and severe. The system was evaluated using 1768 images and achieved cloud-based and real-time processing capabilities. **Results:** The system demonstrated high accuracy (98.34%) in real-time detection with precision (98.22%), sensitivity (99.48%), and specificity (98.22%). Cloud-based processing achieved slightly lower but robust results, with an accuracy of 96.38%. The average processing time was 0.15 s per image, showcasing its real-time potential. **Conclusions:** This automated system provides a scalable and cost-effective solution for the early detection of digital clubbing, enabling timely intervention for lung and cardiovascular diseases. Its high accuracy and real-time capabilities make it suitable for both clinical and home-based health monitoring.

## 1. Introduction

Lung and cardiovascular diseases are two of the leading causes of mortality worldwide, contributing to a significant global health burden. Research has increasingly shown that chronic pulmonary inflammation, whether localized or systemic, is closely associated with both lung and cardiovascular conditions [1]. Despite their prevalence, the early detection of these diseases remains difficult due to the subtle and often asymptomatic nature of their initial stages. This delayed diagnosis frequently leads to disease progression, making these conditions more complex and harder to treat [2,3]. One of the few early and visible clinical signs of both lung and cardiovascular diseases is digital clubbing, as demonstrated in studies by Divani and Arora et al. [4]. Digital clubbing is marked by a bulbous enlargement of the fingertips, a condition typically linked to a range of cardiopulmonary diseases, including lung cancer, chronic obstructive pulmonary disease (COPD), cyanotic congenital heart disease, and idiopathic pulmonary fibrosis [5]. The pathophysiology of clubbing is thought to involve the release of platelet-derived growth factor (PDGF) and vascular endothelial growth factor (VEGF), which contribute to its manifestation and make it a valuable clinical marker for the early detection of cardiopulmonary diseases. Toovey and Eisenhauer have emphasized the significance of clubbing in chronic lung diseases and cyanotic heart conditions, underscoring its diagnostic value [6]. Hans and David further stressed that digital clubbing not only serves as a critical indicator of lung diseases but can also reflect the progression or resolution of the disease over time, making it a dynamic clinical marker [7]. The early detection of this symptom is essential, as timely medical intervention can prevent the worsening of underlying conditions. However, subjective physical examinations alone are often insufficient for accurately detecting clubbing, especially in its early stages, which may result in patients overlooking the severity of their condition and delaying medical consultations [8]. This highlights the need for an accessible, automated detection system that allows users to perform self-assessments. Such a system would enable individuals to monitor their condition and seek timely medical advice, ultimately reducing the risk of disease progression.

In recent years, deep learning has emerged as a transformative tool in medical image processing, offering significant advancements in disease detection and diagnosis [9,10,11,12]. Among various deep-learning techniques, convolutional neural networks (CNNs) have dominated the field of computer vision since 2020, becoming the preferred method for processing medical images due to their superior performance in identifying intricate patterns [13]. Pawan et al. developed an incremental modular network (IMNet) based on the CNN architecture, successfully applying it to both blurred and clear medical images of diseases such as malaria, diabetic retinopathy, and tuberculosis. Their work demonstrated that CNN-based models not only enhance diagnostic accuracy but also improve the speed of detection, ultimately enabling early intervention and better patient outcomes [14]. Ross et al. introduced the Region-Based Convolutional Neural Network (R-CNN), which integrates both the CNN and Recurrent Neural Network (RNN) architectures. This hybrid approach leverages the strengths of CNNs for spatial feature extraction and RNNs for sequential data processing, resulting in superior detection accuracy [15]. In addition to R-CNN, the You Only Look Once, Version 8 (YOLOv8) model has gained recognition as an advanced anchor-based object detection algorithm [16,17,18,19]. Rahul et al. successfully applied YOLOv8 in the rapid detection and classification of skin diseases by utilizing its high-speed image processing capabilities, allowing for the early identification of dermatological conditions without compromising accuracy [20]. This balance between detection speed and precision is crucial for medical applications, as it enhances the efficiency of disease diagnosis and supports timely clinical intervention [21]. In this study, we employ the YOLOv8 model for the initial detection of digital clubbing by precisely identifying the key characteristic points of clubbed fingers in captured images. This model’s robust object detection capabilities allow for rapid and accurate identification, ensuring the reliable recognition of early signs. In addition, we integrate the U-Net deep-learning model, a widely adopted architecture for medical image segmentation, to further analyze the detected areas and provide a preliminary assessment of the severity. U-Net is particularly well-suited for medical image segmentation tasks due to its unique ability to capture both fine details and broader contextual information, making it an ideal choice for this application [22,23]. The U-Net model differs from traditional neural networks in that it exclusively uses convolutional layers, without fully connected layers. Each convolutional layer is followed by a Rectified Linear Unit (ReLU) activation function, which enhances the model’s ability to capture relevant features. This architecture ensures that every pixel in the segmented image retains important contextual information from the original input [24,25,26]. U-Net’s capability has been demonstrated in various medical applications. For instance, Prasad et al. applied U-Net to chest CT images for the early screening of COVID-19, achieving high accuracy in detecting relevant features [27]. Similarly, Reza et al. utilized U-Net to segment diseased areas in skin images, successfully isolating key features from lesions and thereby enhancing classification accuracy [28].

Drawing on the insights from the literature, the primary objective of this study is to develop an automated detection system for digital clubbing, powered by deep-learning models. This system is designed to offer users a quick, straightforward method for detecting digital clubbing, while also enabling the continuous monitoring of the condition. To achieve this, we employ the ESP32-CAM development board in combination with an ultrasonic sensor module and other essential hardware components. By positioning the user’s finger in a predefined marked area, the system ensures consistency in image capture, which is crucial for maintaining the accuracy of the subsequent deep-learning-based recognition process. To achieve the reliable recognition of digital clubbing, this study integrates the YOLOv8 deep-learning model for object detection with the U-Net model for image segmentation [24,29]. By combining these two models, we create a robust system capable of accurately detecting clubbing in the captured images and conducting a preliminary assessment of its severity. To enhance the precision of severity evaluation, we also incorporate the Lovibond angle detection theory [30], a widely recognized method for measuring the angular changes in the nail base—a key indicator of clubbing severity. This led to the development of the Clubbed Fingers Severity Analysis (CFSA) algorithm, which enables the system to categorize the condition into various severity levels. By applying the CFSA algorithm, users can gain a detailed understanding of the severity of their condition, which could prompt timely medical consultation and intervention.

Building on the concepts discussed above, we propose that the developed method for the preliminary detection of digital clubbing offers users an efficient tool to quickly and accurately determine the presence of this condition. This system not only benefits users but also allows family members and healthcare providers to closely monitor the patient’s health, potentially slowing the progression of symptoms and mitigating the risks associated with advanced lung and cardiovascular diseases. To ensure the models used in this study were appropriate for the task, a thorough evaluation and analysis were conducted, focusing on their application and selection rationale. These models were compared against other deep-learning approaches, allowing for a detailed understanding of their strengths and limitations. The results of these comparisons demonstrate that the integrated YOLOv8 and U-Net models outperform existing solutions in detecting digital clubbing, offering superior accuracy and efficiency.

In addition to model evaluation, we also assessed the system’s overall performance by comparing it to similar products on the market, analyzing the differences in preliminary detection capabilities. These assessments serve to improve the system’s robustness and completeness. The ultimate goal of this research is to provide users with a simple and rapid method for the preliminary self-assessment of digital clubbing, facilitating early medical intervention when necessary. We will also investigate other physiological factors contributing to digital clubbing to enhance the system’s diagnostic capabilities. The key contributions of this study include the successful integration of the YOLOv8 and U-Net models into a unified system, enabling the efficient preliminary detection of digital clubbing. Moreover, the performance of the proposed models has been shown to surpass that of other commonly used methods, highlighting the system’s potential as an effective diagnostic tool.

## 2. Materials and Methods

**Figure 1:**
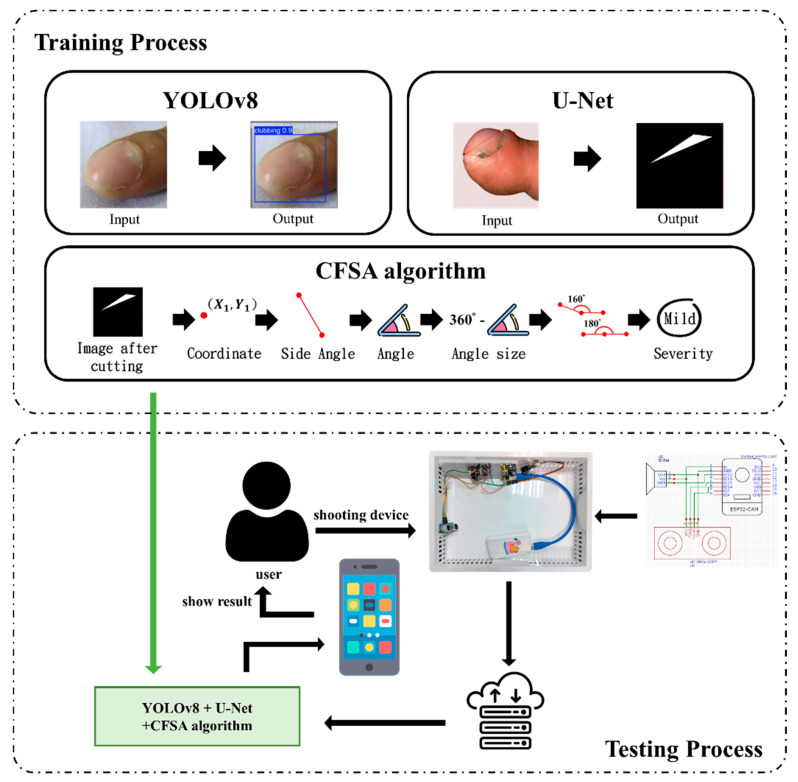
Architecture of the proposed system for detecting clubbed fingers, used for preliminary identification of clubbed fingers.

Figure 1 provides a detailed illustration of the proposed system architecture, encompassing both training and testing phases. The system integrates the YOLOv8 object detection model and the U-Net segmentation model to identify the presence of digital clubbing and conduct an initial severity assessment. Throughout both phases, these models are executed on a personal computer (PC) to optimize computational efficiency and model performance.

To initiate the preliminary detection process, users follow instructions through a custom-designed mobile app, which guides them in using the clubbed fingers detection device. This device captures images, and, upon successful capture, the system indicates completion with two flashes. The captured images are then uploaded to the system where they are processed by the YOLOv8 and U-Net models for recognition and assessment. Subsequently, the Clubbed Fingers Severity Analysis (CFSA) algorithm is applied to determine the severity level, and the results are promptly displayed on the mobile app for user convenience.

### 2.1. Image Acquisition

The objective of this study is to accurately identify and classify the severity of digital clubbing into four distinct levels: normal, mild, moderate, and severe. To achieve this, the study utilizes an open-source image dataset sourced from a GitHub repository, which contains multiple anonymized finger images of uniform dimensions [31]. This dataset originally consisted of 221 images; however, to improve the robustness of the results, the sample size was increased to 400 images by incorporating additional data from publicly available sources and enhancing the diversity of the dataset. The expanded dataset now includes 80 images for testing, 160 for training, and 160 for validation. No additional medical experiments were conducted for data collection as the study solely relied on the augmented dataset. To further enhance model performance, image augmentation techniques such as rotation and Gaussian blur were applied using OpenCV [32]. These augmentations improve the model’s robustness, allowing it to recognize clubbing from different angles and under varying conditions.

The image rotation technique ensures that the model can accurately recognize digital clubbing, regardless of the orientation of the fingers in the captured images. Additionally, Gaussian blur is applied to the rotated images, creating blurred versions that simulate real-world variations in image clarity. This combination of rotation and blur strengthens the model’s ability to detect digital clubbing under a wide range of conditions, including different angles and pixel densities.

Following the application of these augmentation techniques, the total number of images increased to 1768. This expanded dataset was then split into three subsets: 80% for training (1414 images), 10% for testing (177 images), and 10% for validation (177 images). This division ensures that the model is thoroughly trained and evaluated on a diverse range of images, improving its generalization capability across different scenarios.

### 2.2. YOLOv8-Based Model with Deep Feature Extraction

To perform the preliminary identification of digital clubbing in finger images, the YOLOv8 deep-learning model is utilized for initial detection. In addition to YOLOv8, we incorporated deep features extracted from a pretrained convolutional neural network (CNN) such as ResNet to enhance the system’s feature extraction capability. These deep features provide a rich representation of the input images by capturing high-level patterns which, when combined with the traditional structural features extracted by YOLOv8, improve the model’s classification performance. To validate the impact of integrating deep features on model performance, we conducted a series of experiments comparing the classification accuracy using traditional features alone versus the combined deep and traditional features. The results of these experiments indicate a significant improvement in performance metrics, highlighting the effectiveness of incorporating deep features into our detection system. This hybrid approach leverages the advantages of both deep and traditional features, contributing to more accurate and robust disease detection. YOLOv8 is known for its high efficiency in object detection and image classification tasks, offering both speed and accuracy. In this study, YOLOv8 was selected due to its excellent performance in previous medical imaging applications where it has demonstrated fast inference times and precise detection, especially on high-performance computing environments like Amazon EC2 C6i instances [33]. The YOLOv8 architecture is an evolution of the earlier YOLOv5 model, introducing several enhancements, including modifications to the C2f module. These updates allow YOLOv8 to extract more detailed features and contextual information from images, thereby improving its overall detection accuracy [34,35]. This makes it particularly well-suited for identifying the subtle visual signs associated with digital clubbing.

**Figure 2:**
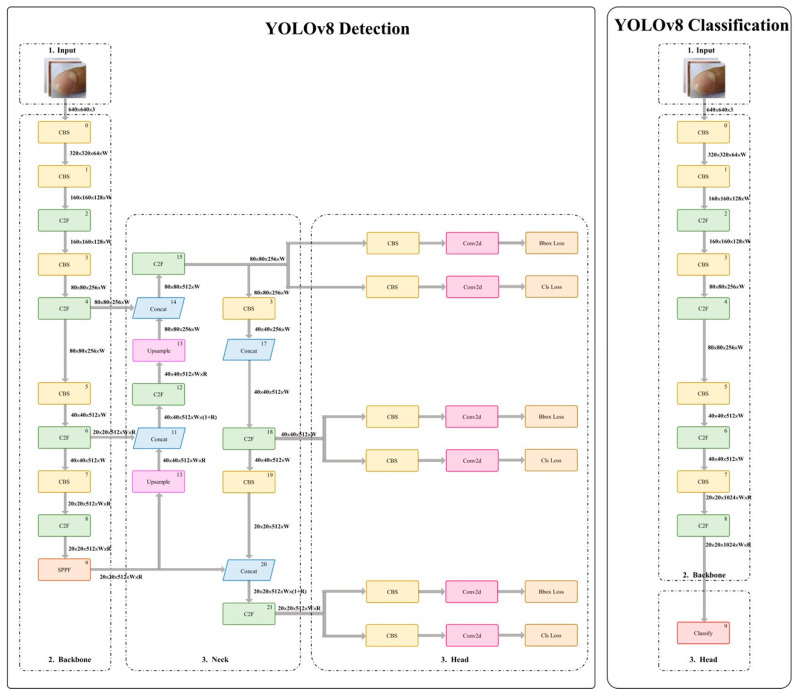
Architecture of the YOLOv8-based model.

Figure 2 illustrates the architecture of the YOLOv8 model, which is composed of two primary components: the backbone (encoder) and the head (decoder). The backbone, similar to traditional Convolutional Neural Networks (CNNs), consists of a series of convolutional layers combined with Conv2d, batch normalization (batchNorm2d), and Sigmoid Linear Unit (SiLU) activation functions. This structure allows the model to extract key features from the input images efficiently. A distinguishing feature of YOLOv8 is the C2f module, which replaces the C3 module found in YOLOv5. The C2f module enhances feature extraction by using fewer parameters, thus improving computational efficiency while preserving the richness of the extracted feature maps. During processing, input images are down-sampled with a stride of 2 to reduce their spatial dimensions, and the Neck component further refines these features, enhancing detection accuracy and model robustness.

The head, or decoder, in the YOLOv8 model up-samples the feature maps generated by the backbone through UpConv2×2 operations, effectively restoring the original resolution of the input image. These up-sampled feature maps are then concatenated with the corresponding feature maps from the backbone, allowing for the integration of high-resolution details with low-resolution contextual information. This ensures that both fine details and broader image features are preserved during detection. The Neck component generates three distinct feature maps (T1, T2, and T3) at varying resolutions, which are then passed to the decoder for further processing. The decoder incorporates three Decoupled-Heads, each consisting of four 3 × 3 convolutional layers and two 1 × 1 Conv2d layers. These heads analyze the feature maps to predict object locations and class probabilities. An anchor-free mechanism is employed, and candidate boxes undergo Region of Interest (ROI) Pooling and convolution operations to produce a final tensor, indicating the probability distribution for each class across the image. 

In the final step of the YOLOv8 detection process, a Conv1×1 layer is applied with non-maximum suppression (NMS) to refine the output. This layer reduces the feature map to the desired number of channels and filters out overlapping bounding boxes, retaining only the most relevant detections. Table 1 details the architecture of the YOLOv8 model, including the resolution and channel numbers for each layer. Each row corresponds to a specific stage (i), providing information on the number of layers (Li), input resolution 〈$\hat{H_{i}}\times \hat{W_{i}}$〉, and output channels $\hat{C_{i}}$. Table 2 outlines the hyperparameters used in all experiments, where the AdamW optimizer is employed with a learning rate of 1 × $10^{-3}$, a batch size of 20, and 150 epochs. These hyperparameters were selected to balance model performance and training efficiency.

The total loss function ($L_{bbox}$) is defined by combining the boundary box regression loss ($L_{cls}$), object classification loss ($L_{conf}$), and object confidence loss ($L_{total}$), to validate the performance of the YOLOv8 model.

(1) $$L_{bbox}=\sum_{i}SmoothL1(\hat{b_{i}}-b_{i})$$

here, $\hat{b_{i}}$ represents the predicted bounding box, while $b_{i}$ represents the ground truth bounding box.

(2) $$L_{cls}=-\sum_{i}\left(y_{i}log\left(\hat{y_{i}}\right)+(1-y_{i})log(1-\hat{y_{i}})\right)$$

here, $y_{i}$ represents the ground truth class label, while $\hat{y_{i}}$ represents the predicted class probability.

(3) $$L_{conf}=-\sum_{i}\left(o_{i}log\left(\hat{o_{i}}\right)+(1-o_{i})log(1-\hat{o_{i}})\right)$$

here, $\hat{o_{i}}$ represents the ground truth confidence (whether the object exists), while $o_{i}$ represents the predicted confidence.

(4) $$L_{total}=\lambda_{bbox}L_{bbox}+\lambda_{cls}L_{cls}+\lambda_{conf}L_{conf}$$

here, $\lambda_{bbox}$, $\lambda_{cls}$, and $\lambda_{conf}$ are the corresponding weight hyperparameters used to adjust the influence of each component of the loss on the total loss, and are set to 1.

### 2.3. U-Net-Based Model

**Figure 3:**
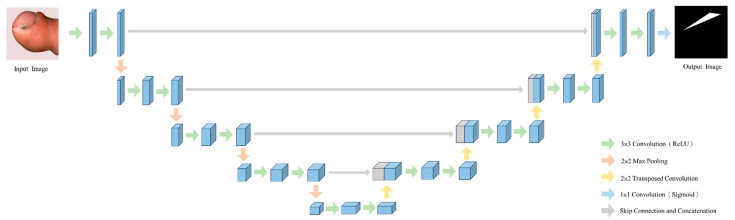
Architecture of the U-Net-based model.

To assess the severity of digital clubbing, the U-Net deep-learning model is employed for image segmentation, enabling the system to perform a detailed analysis of the affected areas. U-Net has proven to be one of the most effective models for image segmentation, particularly in medical applications, due to its ability to capture both high-level contextual information and fine-grained details. Its robust performance in these tasks has been demonstrated by its victory in the ISBI 2015 Challenge, establishing U-Net as a leading choice for biomedical image segmentation tasks [36,37,38]. The architecture of the U-Net model used in this study is illustrated in Figure 3. The U-Net architecture, as its name suggests, follows a U-shaped structure and consists of two key components: the contraction path (encoder) and the expansion path (decoder). The contraction path functions similarly to a traditional Convolutional Neural Network (CNN) and is designed to progressively reduce the spatial dimensions of the input image while extracting increasingly abstract features. This is achieved through a series of blocks, each containing two 3 × 3 convolutional layers followed by a Rectified Linear Unit (ReLU) activation function and a 2 × 2 max-pooling layer with a stride of 2. By reducing the spatial dimensions at each step, the model can capture deeper features critical for accurate segmentation. The expansion path is the core of U-Net’s unique architecture, allowing the model to reconstruct high-resolution feature maps from the compressed representations generated by the contraction path. At each stage of the expansion path, feature maps are up-sampled using 2 × 2 up-convolution (UpConv) layers, which help restore the spatial dimensions of the original input. These up-sampled maps are then concatenated with the corresponding feature maps from the contraction path, ensuring that both low-level and high-level features are preserved during reconstruction. Following the concatenation, the combined feature maps undergo further processing through two 3 × 3 convolutional layers and ReLU activations, enhancing the model’s ability to localize features accurately. Finally, a 1 × 1 convolution layer is used to adjust the number of output channels, producing the final segmentation map that distinguishes between the different severity levels of digital clubbing. 

Table 3 outlines the architecture details of the U-Net model, including the input resolution and the number of channels at each layer. For this study, the input images are sized at 256 × 256 pixels with 3 channels (RGB format), and the table describes the number of layers (Li), input resolution 〈$\hat{H_{i}}\times \hat{W_{i}}$〉, and the output channels $\hat{C_{i}}$ for each stage. Table 4 presents the hyperparameters used for training the U-Net model. The Adam optimizer was selected with a learning rate of 1 × $10^{-3}$, a batch size of 16, and 100 training epochs to ensure effective learning without overfitting. The loss function employed is a combination of binary cross-entropy loss ($L_{CE}$) and Dice loss ($L_{Dice}$), with a weighting factor λ applied to balance the two components. The total loss function ($L_{T}$) helps validate the model’s performance by ensuring both accuracy and smoothness in the segmentation output.

(5) $$L_{BCE}=-\frac{1}{N}\sum_{i=1}^{N}\left[y_{i}\log\left(p_{i}\right)+(1-y_{i})log(1-p_{i})\right]$$

where *N* is the total number of pixels, $y_{i}$ is the true label of the *i*-th pixel (0 or 1), and $p_{i}$ is the predicted probability of the *i*-th pixel.

(6) $$L_{Dice}=1-\frac{2\sum_{i=1}^{N}p_{i}y_{i}}{\sum_{i=1}^{N}p_{i}+\sum_{i=1}^{N}y_{i}}$$

where $p_{i}$ is the predicted value of the *i*-th pixel and $y_{i}$ is the true label of the *i*-th pixel.

(7) $$L_{T}=L_{BCE}+\lambda L_{Dice}$$

where λ is the weight coefficient that adjusts the relative importance of BCE and Dice loss, set to 1 to balance their influence.

### 2.4. Clubbed Fingers Severity Analys (CFSA) Algorithm

To effectively differentiate between various severity levels of digital clubbing, this study incorporates the Lovibond angle detection method as originally proposed by John L. [30]. This method relies on visually assessing the angle formed by three anatomical points: the nail matrix, the first phalanx nail fold, and the nail plate. Based on these measurements, a severity rating system was developed to classify the condition into four distinct categories [39]:

- Normal: when the measured Lovibond angle is approximately 160°;
- Mild: when the angle falls between 160° and 180°;
- Moderate: when the angle is exactly 180°;
- Severe: any angle greater than 180°.

This rating system not only aids in the detection of clubbing but also provides actionable insights into the severity of the condition, enabling healthcare professionals to prioritize cases based on urgency. The CFSA algorithm automates this process, allowing users to receive a clear and detailed severity classification during self-assessment.

**Figure 4:**
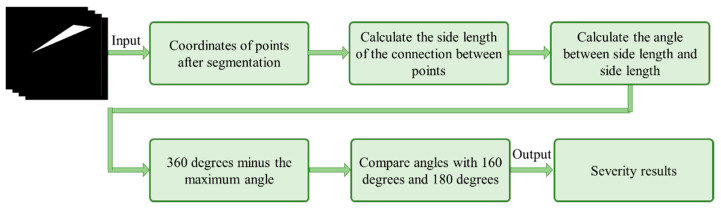
Diagram illustrating the proposed CFSA algorithm.

While visual assessment is straightforward, it can be difficult to accurately differentiate between asymptomatic, mild, and moderate cases of digital clubbing, leading to potential delays in diagnosis and treatment. To address this challenge, we expand on the Lovibond angle detection theory by incorporating the segmented images produced by the U-Net model into a custom Clubbed Fingers Severity Analysis (CFSA) algorithm. This algorithm automates the process of determining the severity of clubbing, reducing the subjectivity associated with visual inspection. As depicted in Figure 4, the CFSA algorithm works by extracting three critical feature points from the U-Net segmentation output: the nail matrix, the first phalanx nail fold, and the nail plate. The coordinates of these points are used to form a triangle. By applying the cosine formula, we can accurately calculate the lengths of the triangle’s sides and, consequently, determine the angles. This mathematical approach provides a precise and objective method for assessing the severity of clubbing based on well-defined geometric principles. Once the three angles of the triangle are calculated, the largest angle is subtracted from 360° to derive the final detection angle required for the Lovibond angle method. This angle is then used to classify the condition into mild, moderate, or severe stages of digital clubbing, in accordance with the thresholds established by the Lovibond detection method. Fingers that are determined to be asymptomatic or normal during the initial YOLOv8 classification process are not subjected to further severity assessment. This automated approach allows for precise, quantitative identification of severity levels, offering users a clear and accurate understanding of their condition. By providing specific numeric outputs, the system makes it easier for users to distinguish between the various stages of digital clubbing, facilitating timely medical consultation when needed.

(8) $$\bar{AB}=\sqrt{{\left(x_{B}-x_{A}\right)}^{2}+{\left(y_{B}-y_{A}\right)}^{2}}$$

(9) $$\bar{AC}=\sqrt{{\left(x_{C}-x_{A}\right)}^{2}+{\left(y_{C}-y_{A}\right)}^{2}}$$

(10) $$\bar{BC}=\sqrt{{\left(x_{C}-x_{B}\right)}^{2}+{\left(y_{C}-y_{B}\right)}^{2}}$$

**Figure 5:**
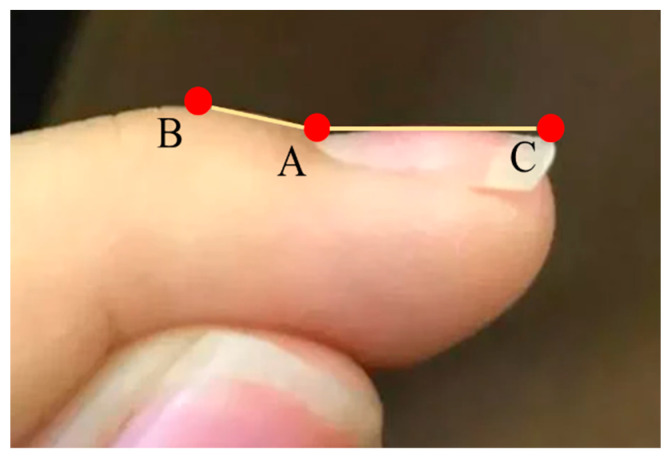
The three feature points required for the Lovibond angle measurement are marked as follows: Point A is the nail base, Point B is the first phalanx nail fold, and Point C is the nail plate.

Let the nail matrix, the first phalanx nail fold, and the nail plate be denoted as points A, B, and C (Figure 5), respectively, with coordinates A($x_{A},y_{A}$), B($x_{B},y_{B}$), and C($x_{C},y_{C}$).

(11) $$\cos(\theta )=(\frac{AB^{2}+AC^{2}-BC^{2}}{2\times AB\times AC})$$

(12) $$\theta =arccos(\frac{AB^{2}+AC^{2}-BC^{2}}{2\times AB\times AC})$$

**Figure 6:**
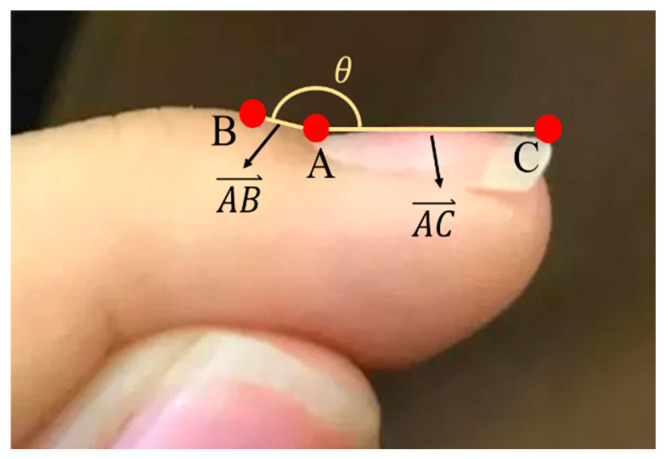
The angle formed by the lines connecting the three points—nail base, first phalanx nail fold, and nail plate—is described as follows: Point A is the nail base, Point B is the first phalanx nail fold, and Point C is the nail plate. The vector $\overset{⃑}{AB}$ represents the direction from the nail base to the first phalanx nail fold, and the vector $\overset{⃑}{AC}$ represents the direction from the nail base to the nail plate.

The angles of the triangle can be determined using the cosine formula. Equations (11) and (12) demonstrate the calculation of ∠BAC. To compute ∠ABC and ∠ACB, the same method is applied (Figure 6).

(13) $$\mathrm{Remaining}\;\mathrm{Angle}=360{}^{\circ}-\max(\theta_{A},\theta_{B},\theta_{C})$$

where max($\theta_{A}$,$\theta_{B}$,$\theta_{C}$) is the largest of the three angles.

### 2.5. Clubbed Fingers Detection Device

**Figure 7:**
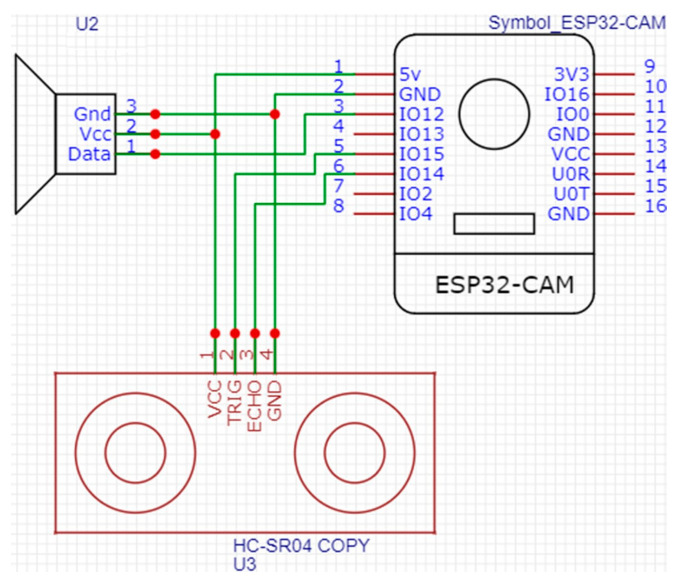
ESP32-CAM development board.

The clubbed fingers detection device is specifically designed to capture high-quality images of the user’s fingers for analysis. The core of this imaging device is the ESP32-CAM development board, which is integrated with several essential hardware components, including an ultrasonic sensor module, a passive buzzer, and an OV2640 2MP camera module, as shown in Figure 7. The ultrasonic sensor plays a crucial role in ensuring accurate image capture by measuring the distance between the finger and the camera. When the finger is placed within the correct range, the passive buzzer emits a prompt, signaling to the user that the finger is properly positioned for imaging. This ensures that all images are captured consistently, which is critical for maintaining the accuracy of subsequent image processing. The image capture process is initiated when the user places their finger in the designated position on the device. The ultrasonic sensor continuously monitors the distance between the finger and the camera, ensuring that it falls within the optimal range of 21 to 25 cm. If the finger is correctly positioned, the passive buzzer alerts the user, and the image capture sequence begins. During this process, the device’s flash blinks three times: first, when the ESP32-CAM initiates the capture, second, when the image capture is complete, and, finally, when the captured image is successfully uploaded to the cloud. These visual indicators provide feedback to the user, ensuring that they are aware of each step in the process.

The system’s hardware costs include the ESP32-CAM development board ($10), ultrasonic sensor ($3), passive buzzer ($1), and camera module ($5), resulting in a total hardware cost of approximately $19. The software used in this study is based on open-source platforms like PyTorch, which incurs no additional software expenses. Compared to commercial systems for medical image analysis, which can range from $500 to $2000, our solution is significantly more affordable, offering a viable option for resource-limited settings.

### 2.6. Performance

The performance of the proposed system is evaluated using a confusion matrix, as shown in Table 5. The confusion matrix provides a comprehensive overview of the relationship between the actual and predicted classes in the test set. In this matrix, each row represents the actual class, while each column represents the predicted class. Based on the system’s detection results, a sample is categorized as True Positive (TP) if the system correctly identifies the presence of digital clubbing. A False Positive (FP) sample occurs if the system incorrectly identifies digital clubbing when it is not present. Similarly, a True Negative (TN) sample indicates that the system correctly rejected a sample without digital clubbing, while a False Negative (FN) sample represents a missed detection where digital clubbing was present but not identified by the system. This classification provides insight into the system’s accuracy, specificity, sensitivity, and precision, which are critical metrics for evaluating the overall performance in detecting digital clubbing at different severity levels. 

To comprehensively evaluate the performance of the proposed model, several key metrics are employed, including accuracy, sensitivity, specificity, and precision. These metrics are defined mathematically and are essential for understanding how well the system performs in different scenarios, particularly in terms of its ability to accurately detect and differentiate between normal and clubbed fingers at varying severity levels. The following Equations (14)–(17) provide the formal definitions of these performance metrics.

(14) $$\mathrm{Accuracy}=\frac{TP+TN}{TP+TN+FP+FN}$$

(15) $$\mathrm{Sensitivity}=\frac{TP}{TP+FN}$$

(16) $$\mathrm{Specificity}=\frac{TN}{TN+FP}$$

(17) $$\mathrm{Precision}=\frac{TP}{TP+FP}$$

## 3. Results

The deep-learning models used in this study, including YOLOv8 for object detection and U-Net for image segmentation, were trained, validated, and tested on a computer with the following specifications: an 8-core Intel i5-12400 CPU, 16GB of RAM, an Nvidia RTX 2050 8GB GPU, and CUDA version 7.X. PyTorch served as the primary framework for the implementation and execution of the models.

**Figure 8:**
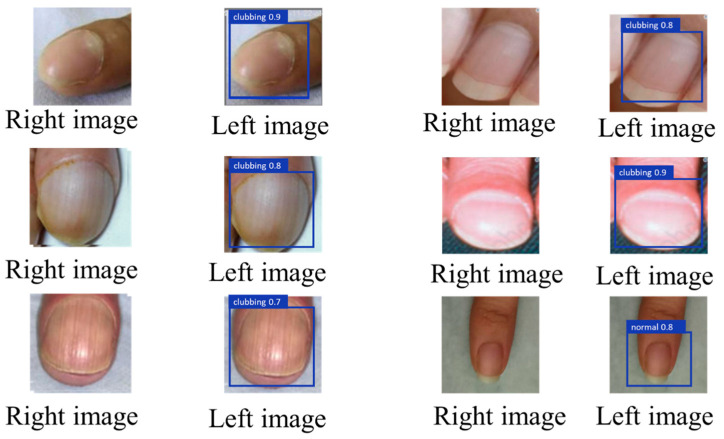
Before inputting the annotated image into YOLOv8 (**left** image) and after YOLOv8 recognition (**right** image).

**Figure 9:**
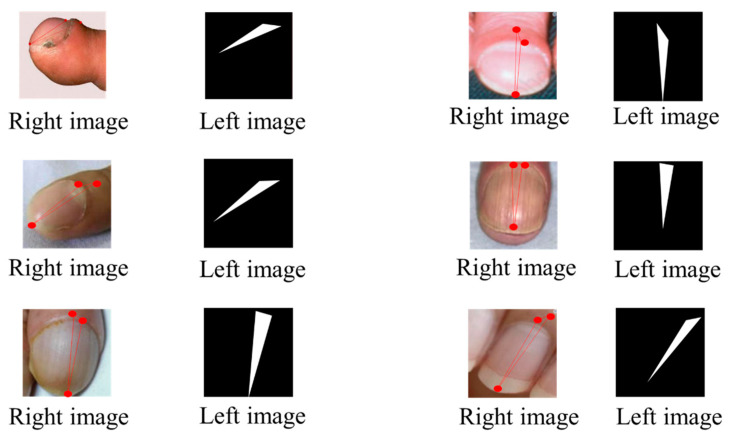
The annotated image before inputting into U-Net (**left** image) and the output after U-Net segmentation (**right** image).

In addition to identifying the presence of digital clubbing, the system implemented a severity rating system that categorizes the condition into four levels: normal, mild, moderate, and severe. Figure 8 and Figure 9 present the visual outputs from the YOLOv8 classification and U-Net segmentation processes, respectively, with the severity level clearly indicated for each detected case. This rating system provided actionable insights, allowing healthcare professionals or patients to understand the urgency of their condition based on the severity classification.

Table 6 provides the confusion matrix for real-time computations [40,41,42], illustrating the performance of the integrated YOLOv8 and U-Net models as a single system for digital clubbing detection. The results indicate that the system achieved an impressive overall accuracy of 98.34%, with a precision of 98.22%, sensitivity of 99.48%, and specificity of 98.22%. These high performance metrics reflect the system’s robustness in accurately identifying digital clubbing, including differentiating between different severity levels, while maintaining a high degree of precision and reliability in real-time operations.

To assess the robustness of the integrated YOLOv8 and U-Net models in challenging environments, the system’s performance was evaluated under conditions of salt-and-pepper noise. This type of noise introduces random pixel variations, with some pixels altered to their maximum value (white “salt”) and others to their minimum value (black “pepper”), mimicking real-world image distortions and disturbances. The number of test samples in this experiment matched those used in the real-time calculations, ensuring consistency across different testing conditions. This evaluation aimed to determine how effectively the system could maintain its high detection accuracy and precision in the presence of image noise, further validating its potential for use in varied and noisy environments.

Table 7 presents the confusion matrix for the integrated YOLOv8 and U-Net models when tested under conditions of salt-and-pepper noise. Despite the introduction of this noise, the system maintained strong performance, achieving an accuracy of 97.12%, precision of 97.41%, sensitivity of 98.52%, and specificity of 97.62%. These results clearly demonstrate the robustness of the proposed detection system in handling noisy environments. 

The slight decrease in performance metrics—specifically, a reduction of 0.22% in accuracy, 0.81% in precision, 0.96% in sensitivity, and 0.60% in specificity—indicates that the system is resilient to noise-induced image degradation. This minimal drop in performance suggests that the integrated YOLOv8 and U-Net models are well-suited for practical use in real-world environments where image quality may vary due to noise or other visual distortions.

In this study, we successfully integrated the trained YOLOv8 and U-Net models into a mobile application, facilitating the real-time and cloud-based detection of digital clubbing. The image-capturing device was built using the ESP32-CAM development board, combined with essential hardware components including the OV2640 2MP camera module. This configuration allows the system to capture high-resolution images of the user’s fingers and upload them directly to the cloud for storage and processing. Once an image is captured, the trained YOLOv8 and U-Net models immediately process the most recently stored image in the cloud, ensuring timely analysis. The results are then displayed on the system interface, providing users with real-time feedback on the presence and severity of digital clubbing. The same number of test samples used in real-time calculations was applied to evaluate the cloud-based computations, ensuring consistent performance comparisons between the two methods.

Table 8 presents the confusion matrix for cloud-based calculations using the integrated YOLOv8 and U-Net models. The system achieved an accuracy of 96.38%, precision of 97.24%, sensitivity of 97.64%, and specificity of 96.61% in cloud-based operations. When comparing these results to the real-time performance shown in Table 6, the cloud-based system’s performance remains very close, with only slight decreases in accuracy, precision, sensitivity, and specificity by 1.96%, 0.98%, 1.84%, and 1.61%, respectively. These results confirm that the integration of YOLOv8 and U-Net into both real-time and cloud-based systems delivers consistently high performance, maintaining robustness and accuracy across different operating environments.

To evaluate the effectiveness of incorporating deep features into the system, we conducted a series of experiments comparing the following configurations in Table 9: using only traditional features (YOLOv8), using deep features (ResNet), and combining both types of features.

The results indicate that incorporating deep features, especially when combined with traditional features, improves the model’s overall performance. The combined features configuration achieves the highest accuracy, precision, sensitivity, and specificity, demonstrating the effectiveness of this approach in classifying digital clubbing.

## 4. Discussion

In this study, the YOLOv8 model was employed for the classification of digital clubbing in finger images, while the U-Net model was used for image segmentation, enabling a preliminary assessment of the severity of the condition. To evaluate the effectiveness of these models, we conducted a series of comparisons with other existing deep-learning models used for similar medical applications. These comparisons aimed to determine whether our integrated system outperforms alternative approaches in terms of detection accuracy, speed, and precision. Furthermore, we assessed the accuracy of the finger positioning mechanism in our photography setup, comparing it to similar detection systems available in the market. The consistency in finger placement was crucial for achieving reliable results, and this aspect of our system showed significant improvement in both accuracy and user experience over other products.

To evaluate the superiority of the YOLOv8 model in image classification, we conducted a performance comparison with other widely used object detection models. Table 10 presents the results of this comparison, demonstrating that YOLOv8 achieves an overall accuracy of 95.2%, a mean Average Precision (mAP) of 90.3%, and a recall rate of 93.5%. These metrics place YOLOv8 ahead of competing models in terms of accuracy, precision, F1 score, and recall rate. Notably, YOLOv8 ranked second only to Faster R-CNN in terms of average precision and second to the SSD model in inference time. These results clearly indicate that YOLOv8 offers a significant advantage over other models in detecting digital clubbing, with superior performance across multiple evaluation criteria. The model’s ability to balance speed and accuracy makes it particularly suitable for real-time medical image classification tasks.

To determine if the YOLOv8 model used in this study performs better than other versions of YOLO, images of clubbed fingers were input into different versions of YOLO models for comparison. This comparison included YOLOv8 and the four preceding versions closest to YOLOv8. Table 11 displays the performance comparison results of the YOLOv8 model with other versions. From the comparison data, it can be observed that the YOLOv8 model achieved a mean Average Precision (mAP) of 90.3%, an inference time of 12 ms, and 76 FPS. The results indicate that using YOLOv8 for the image classification task of clubbed fingers in this study outperforms other YOLO series versions.

The comparison results of YOLOv8’s image classification performance between these two methods indicate that the model used in this study outperforms other models in image recognition. It demonstrates excellent performance in image classification, with good accuracy and recall rates.

In addition to the YOLOv8 model, the U-Net model was used in this study for image segmentation, allowing for a detailed preliminary assessment of the severity of digital clubbing. To verify whether U-Net outperforms other segmentation models commonly used in medical imaging, we conducted a comparative analysis by inputting the same set of clubbed finger images into multiple segmentation models. The results were then analyzed to evaluate U-Net’s performance in terms of segmentation accuracy, precision, and computational efficiency. This comparison demonstrated that U-Net not only excelled in segmenting the affected regions of the fingers but also provided an accurate severity classification, reinforcing its effectiveness in medical image segmentation tasks. The model’s ability to capture both high-level and detailed features made it a superior choice for this application. Table 12 shows the performance comparison results of the U-Net model with other models. From these comparison results, it is evident that the U-Net model achieved an mAP of 92.5%, an inference time of 25 ms, and a total number of trainable parameters of 20.5 M for the preliminary assessment of the clubbed fingers severity. The U-Net model outperforms other similar models in terms of the mAP, inference time, parameters, and Dice Score, and its model size and Jaccard Index are only slightly inferior to those of FCN. These results indicate that the U-Net model provides superior segmentation performance for clubbed fingers images compared to other similar models.

In this study, we designed a custom clubbed fingers detection device for capturing images of clubbed fingers and used the YOLOv8 model and U-Net model for the preliminary classification and severity assessment of the condition. The CFSA algorithm is then applied for a preliminary determination of the severity of clubbed fingers, with the results displayed on the system screen. Our goal is to provide a simple and intelligent way for users to easily complete the detection process. Currently, there are no online tools available for preliminary clubbed fingers detection. Therefore, to verify the accuracy and effectiveness of our system, we compared it with existing detection methods. The comparative methods include low-dose computed tomography (CT) [43,44,45,46,47,48,49], Shamroth’s window test [50], and Google DermAssist [51]. The comparison was based on the ISO/IEC 25010:2023 standards [52], evaluating functionality, immediacy, usability, safety, and portability. Table 13 shows the functional comparison results between the proposed detection method and other existing methods. The comparison indicates that our system outperforms other market methods in all evaluated metrics. By integrating deep-learning models into the system, our approach achieves high accuracy in clubbed fingers detection.

The time complexity of the YOLOv8 model is approximately $O(n^{2})$, where nnn represents the number of pixels in the input image. U-Net’s time complexity is similarly $O(n^{2}\cdot d)$, where ddd is the depth of the network. We also measured the runtime performance of our system on a computer with an Intel i5-12400 CPU, 16 GB RAM, and an Nvidia RTX 2050 GPU. The average processing time per image for the YOLOv8 and U-Net combination was approximately 0.15 s, which is competitive in real-time processing applications.

In comparison, other object detection models, such as Faster R-CNN and SSD, showed slower inference times of 0.35 s and 0.25 s per image, respectively. These models, while providing high accuracy, required more computational resources due to their complex architectures. The YOLOv8 model outperformed them in terms of runtime efficiency without sacrificing accuracy, making it a more suitable option for real-time applications in clinical settings. Table 14 summarizes the runtime and time complexity comparisons across different models:

The Clubbed Fingers Severity Analysis (CFSA) algorithm effectively classified the severity of digital clubbing into four categories based on the Lovibond angle. During the evaluation using 1768 images, the CFSA algorithm showed an overall classification accuracy of 96.7% across the four severity levels. Table 15 summarizes the accuracy, precision, and recall for each severity level. The system’s performance in classifying moderate and severe cases was particularly strong, with an accuracy of 98.5% and 97.3%, respectively, while mild cases were classified with a 95.2% accuracy.

## 5. Conclusions

This study presents an innovative integrated system that combines the YOLOv8 object detection model and the U-Net image segmentation model to enable the rapid and accurate preliminary detection of digital clubbing. By leveraging the ESP32-CAM development board and other hardware components, the system captures high-quality images of the user’s fingers for analysis. The YOLOv8 model is employed to detect the presence of digital clubbing, while the U-Net model performs image segmentation to analyze affected areas in detail. To further enhance diagnostic precision, the Clubbed Fingers Severity Analysis (CFSA) algorithm is applied, providing an automated and objective method for classifying the severity of the condition into normal, mild, moderate, or severe categories. This integration of advanced models and hardware allows users to obtain real-time assessments, facilitating early medical intervention and the continuous monitoring of their condition.

The performance of the system was rigorously tested using an open-source dataset obtained from GitHub. During real-time computations, the system achieved a high accuracy of 98.34%, while cloud-based computations demonstrated an accuracy of 96.38%. These results highlight the robustness, reliability, and practical applicability of the system in accurately detecting and assessing the severity of digital clubbing. The combination of high precision, sensitivity, and specificity makes this system a valuable tool for preliminary screenings, particularly in resource-limited settings where real-time diagnostic tools are needed.

However, it is important to acknowledge some limitations in the current study. The training and testing data were sourced exclusively from an open dataset, which may not fully represent the range of imaging conditions found in real-world clinical environments. Factors such as varying lighting conditions, finger positioning, and skin texture were not extensively tested, potentially impacting the clarity and accuracy of captured images in less controlled environments. Additionally, the system has yet to be tested on a wider variety of populations and diverse clinical settings.

The system demonstrates significant potential for the early detection of lung and cardiovascular diseases. In addition to achieving high accuracy, the system was capable of real-time analysis, providing immediate feedback to users. This real-time capability is critical for early screening in clinical and home settings, enabling proactive health management.

Future work will focus on addressing these limitations by expanding the dataset to include images from different populations and environments, allowing the system to be tested under more diverse imaging conditions. This will help to ensure that the system can maintain a high accuracy and reliability in real-world applications. Moreover, additional research will explore incorporating other relevant physiological markers, such as nail bed curvature and skin tone variations, which could further improve the system’s diagnostic capabilities. These enhancements aim to increase the system’s robustness and adaptability, making it a more comprehensive tool for the early detection of digital clubbing and related cardiopulmonary conditions.

In conclusion, the proposed system shows significant promise as a practical and efficient tool for the early detection of digital clubbing, with the potential to improve patient outcomes through timely diagnosis and intervention. Continued development and testing in broader environments will ensure that the system can be effectively deployed in clinical and non-clinical settings, contributing to the better management of lung and cardiovascular diseases.

## Figures and Tables

**Table 1:** For the YOLOv8-based model, stage i has $\hat{L_{i}}$ layers, with an input resolution of $\hat{H_{i}}\times \hat{W_{i}}$ and output channels of $\hat{C_{i}}$.

Stage *i*	Operator $\hat{F_{i}}$	Resolution $\hat{H_{i}}\times \hat{W_{i}}$	Channels $\hat{C_{i}}$	Layer $\hat{L_{i}}$
1	Conv3×3	640 × 640	32	1
2	Conv3×3	320 × 320	64	1
3	C2f3×3	160 × 160	128	2
4	Conv3×3	80 × 80	256	1
5	C2f3×3	40 × 40	512	2
6	SPPF3×3	20 × 20	512	1
7	C2f3×3	40 × 40	256	2
8	Conv3×3	20 × 20	512	1
9	C2f3×3	20 × 20	1024	2

**Table 2:** The hyperparameters for the YOLOv8-based model training.

Hyperparameters	Selected Values
Loss function	$L_{total}=\lambda_{bbox}L_{bbox}+\lambda_{cls}L_{cls}+\lambda_{conf}L_{conf}$
Optimizer	AdamW
Learning rate	1 × $10^{-3}$
Batch size	20
Epoch	150

**Table 3:** For the U-Net-based model with an input image size of 256 × 256 and 3 channels (e.g., RGB images), each stage *i* has $\hat{L_{i}}$ layers, with Input Resolution 〈$\hat{H_{i}}\times \hat{W_{i}}$〉 and Output Channels $\hat{C_{i}}$.

Stage *i*	Operator $\hat{F_{i}}$	Resolution $\hat{H_{i}}\times \hat{W_{i}}$	Channels $\hat{C_{i}}$	Layer $\hat{L_{i}}$
1	Conv3×3	256 × 256	64	2
2	Conv3×3	128 × 128	128	2
3	Conv3×3	64 × 64	256	2
4	Conv3×3	32 × 32	512	2
5	Conv3×3	16 × 16	1024	2
6	Transposed Conv2×2 + Concat	32 × 32	512	3
7	Transposed Conv2×2 + Concat	64 × 64	256	3
8	Transposed Conv2×2 + Concat	128 × 128	128	3
9	Transposed Conv2×2 + Concat	256 × 256	64	3
10	Conv1×1 + Sigmoid	256 × 256	1	1

**Table 4:** The hyperparameters for the U-Net-based model training.

Hyperparameters	Selected Values
Loss function	$L_{T}$ = $L_{BCE}$ + λ$L_{Dice}$
Optimizer	Adam
Learning rate	1 × $10^{-3}$
Batch size	16
Epoch	100

**Table 5:** Confusion matrix.

Estimated Class			
		Positive	Negative
Actual Class	Positive	TP (True Positive)	FN (False Negative)
	Negative	FP (False Positive)	TN (True Negative)

**Table 6:** Confusion matrix for real-time computations based on integrating YOLOv8 and U-Net models into a single system.

	Normal	Mild	Moderate	Severe
Normal	47	4	1	0
Mild	3	29	2	1
Moderate	2	2	34	5
Severe	0	0	3	43

**Table 7:** Confusion matrix for real-time calculations with the integrated YOLOv8 and U-Net models under conditions where some pixel values in the image are altered.

	Normal	Mild	Moderate	Severe
Normal	39	4	2	0
Mild	7	22	4	6
Moderate	4	7	28	9
Severe	1	2	7	35

**Table 8:** Confusion matrix for cloud-based calculations with YOLOv8 and U-Net models integrated into a single system. The number of test samples is the same as in the real-time calculations.

	Normal	Mild	Moderate	Severe
Normal	37	5	2	2
Mild	5	20	6	7
Moderate	5	8	24	9
Severe	1	2	9	32

**Table 9:** Table 9 presents the performance metrics (accuracy, precision, sensitivity, and specificity) for each configuration.

Configuration	Accuracy	Precision	Sensitivity	Specificity
Traditional Features	97.24%	96.45%	98.12%	96.78%
Deep Features (ResNet)	97.89%	97.34%	98.47%	97.11%
Combined Features	98.34%	98.22%	99.48%	98.22%

**Table 10:** Performance comparison results of YOLOv8 model with other models.

Model	Accuracy	Precision	mAP	Inference Time	Model Size	F1 Score	Recall
YOLOv8	95.2%	94.8%	90.3%	12 ms	25 MB	0.92	93.5%
SSD	87.6%	94.8%	85.0%	8 ms	22 MB	0.88	89.1%
RetinaNet	89.5%	88.7%	88.3%	15 ms	30 MB	0.90	90.2%
Faster R-CNN	93.4%	93.0%	91.5%	25 ms	40 MB	0.91	92.7%
EfficientNet	92.1%	90.5%	89.0%	18 ms	20 MB	0.89	91.0%
ResNet	90.7%	89.6%	87.8%	20 ms	35 MB	0.88	91.2%

**Table 11:** Performance comparison results of YOLOv8 model and other versions of the model.

Model	mAP	Inference Time	FPS	Parameters	Input Size
YOLOv8	90.3%	12 ms	76FPS	43.7 M	640 × 640
YOLOv7	71.2%	14 ms	71FPS	37.2 M	640 × 640
YOLOv6	69.1%	15 ms	66FPS	20.1 M	640 × 640
YOLOv5	68.5%	16 ms	62FPS	7.3 M	640 × 640
YOLOv4	65.7%	20 ms	50FPS	64 M	608 × 608

**Table 12:** Performance comparison results of U-Net model with other models.

Model	mAP	Inference Time	Parameters	Model Size	Dice Score	Jaccard Index
U-Net	92.5%	25 ms	20.5 M	38 MB	0.91	0.78
ResUNet	88.1%	30 ms	34.8 M	52 MB	0.85	0.78
V-Net	87.0%	35 ms	32.5 M	48 MB	0.84	0.77
DenseNet	89.2%	40 ms	28.7 M	42 MB	0.86	0.80
SegNet	82.7%	45 ms	29.5 M	40 MB	0.81	0.73
FCN	83.9%	28 ms	25.6 M	35 MB	0.83	0.74

**Table 13:** Functional comparison of the proposed detection method and other detection methods.

	Functionality	Immediacy	Usability	Safety	Portability
Proposed Method	High accuracy in clubbed fingers detection	Effective for internal conditions	Simple but less precise	Limited to skin conditions	Mobile application
Low-Dose CT	Effective for internal conditions	Requires scheduled appointment	Requires medical equipment	Minimal radiation exposure	Requires specialized equipment
Shamroth’s Window Test	Simple but less precise	Immediate with manual assessment	Requires manual practice	Non-invasive	Portable but manual
Google DermAssist	Limited to skin conditions	Depends on image submission time	Requires internet and device	Non-invasive	Mobile application

**Table 14:** Comparison of time complexity, runtime, and accuracy across detection models.

Model	Time Complexity	Average Processing Time (s/Image)	Accuracy (%)
YOLOv8 + U-Net	$O\left(n^{2}\right)+O(n^{2}\cdot d)$	0.15	98.34
Faster R-CNN	$O(n^{3})$	0.35	93.4
SSD	$O(n^{2})$	0.25	87.6

**Table 15:** Severity classification performance using CFSA algorithm.

Severity Level	Accuracy (%)	Precision (%)	Recall (%)
Normal	97.1	96.5	96.8
Mild	95.2	94.9	95.0
Moderate	98.5	97.8	98.2
Severe	97.3	97.1	97.0

## Data Availability

The original contributions presented in the study are included in the article, further inquiries can be directed to the corresponding author.
